# Combined surgical correction of pectus carinatum and juvenile kyphosis

**DOI:** 10.1093/icvts/ivac087

**Published:** 2022-04-05

**Authors:** Bashaer Iwaiwi, Bisanne Hamdi Shaqqura, Alaeddin Sabbah, Firas Emad Abu Akar

**Affiliations:** 1 Medical Research Club, Faculty of Medicine, Al-Quds University, Jerusalem, Palestine; 2 Department of Cardiothoracic Surgery, Al-Makassed Charitable Society Hospital, East Jerusalem, Palestine; 3 Department of Orthopedic Surgery, Al-Makassed Charitable Society Hospital, East Jerusalem, Palestine

**Keywords:** Combined pectus with scoliosis, Abramson's procedure, Minimally invasive correction of pectus

## Abstract

Pectus carinatum may rarely be associated with kyphosis. However, the correlation between both conditions is not well reported. Therefore, there are no reports for combined correction of both deformities in the same patient. Moreover, studies estimating the kyphosis prevalence in patients with pectus carinatum are lacking. To our knowledge, this is the first paper to present such a case. We report an 18-year-old boy with both pectus carinatum and kyphosis that were surgically corrected in a combined procedure. The indication of surgery is cosmetic, and the postoperative recovery included pneumothorax but was otherwise uneventful and satisfactory.

## INTRODUCTION

Pectus carinatum (PC) is a congenital chest wall anomaly with an incidence of 0.06%. It becomes prominent at puberty when the growth is accelerated. Peak age is 18 and 16 years in males and females, respectively.

Usually, it is an isolated anomaly, but it can present with other anomalies; including juvenile kyphosis, an increase in forwarding curvature of the spine, due to a structural deformity of the thoracolumbar spine that usually occurs near puberty with a prevalence of 4–8%. However, this association is extremely rare.

PC can lead to dyspnoea and decreased endurance while kyphosis leads to back pain; both can be corrected surgically or via bracing.

## CASE REPORT

An 18-year-old male presented with a chest protrusion since birth with accelerated worsening of the protrusion and a rapidly progressive kyphosis throughout the last 2 years.

On examination, he had an asymmetrical elevation of the chest wall involving the lower third of the sternum at the second to eighth ribs with thoracolumbar kyphosis with no other significant findings on examination nor history.

Lateral chest radiographs showed abnormal protrusion of the sternum with abnormal curvature of the spine at the thoracic level.

**Figure 1:**
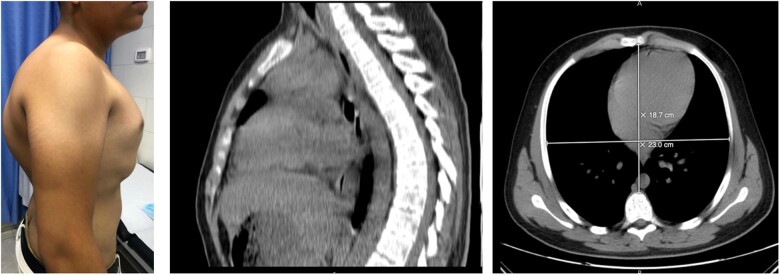
Pre-surgery graphs.

Computed tomography scan of the chest revealed chondrogladiolar type of PC with Haller index of 1.07 and thoracic kyphosis with Cobb’s angle of 90°. Pulmonary function tests and echocardiogram were unremarkable (Fig. 1).

Surgical correction for both deformities simultaneously was planned for cosmetic concerns.

Before the surgery, the patient was admitted to be prepared after signing the consent form. All the preoperative preparations were done including chest computed tomography scan, spine X-ray, echocardiography…etc. Four units of blood and a place in the intensive care unit were prepared.

A multidisciplinary team plan was put by surgeons and anaesthesiologist to be ready for all the scenarios.

The spine procedure was carried by a spine surgeon under general anaesthesia and electrophysical study while the patient is in a prone position. After exposing the vertebrae from T2-L1, 12 pedicular screws were applied on each side, starting from the left side to the right one after identifying the pedicles. Ponte osteotomy was performed at levels of T7-T8 and T8-T9. On the sagittal plane, 2 cross-bridges were applied, and Nippler harvested the spinous processes; later applied as a bone graft. Subsequently, the patient was placed in a supine position to correct PC using the Abramson technique. Re-draping and skin coverage were done, and the bar was measured. Two incisions on the anterior axillary line at the sixth intercostal space were made bilaterally. Two ribs were shaved and surrounded by metal wires. The bar stabilizer was fixed to the wires on both sides. Afterwards, the guide was introduced under the skin, followed by the measured bar fixed to the stabilizer using metal wires and screws. The operation continued for 3 h with a total blood loss of 1100 cc during the surgery.

After the operation, the patient was transferred to intensive care unit for close monitoring with a pain score of 8. The next day, he developed a small left side pneumothorax, which was drained; the chest tube was removed on the third day and the pain score was 3. The patient stayed for 5 days before discharge. The postoperative radiograph showed satisfactory correction of the deformity with the new Cobb’s angle measuring 45°. Moreover, the modified Nuss bar is planned to be removed within 3 years.

**Figure 2:**
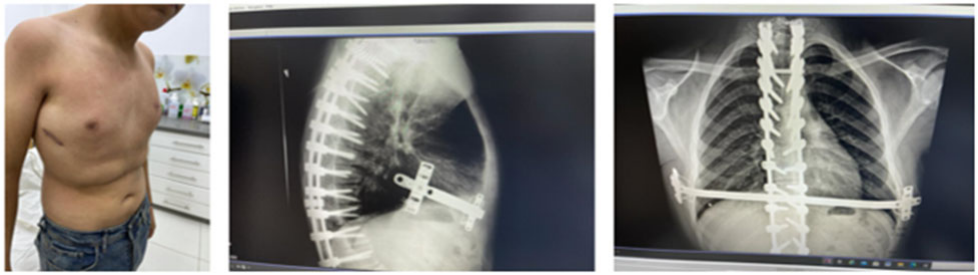
Post-surgery graphs

On follow-up, the results were satisfactory (Fig. 2).

## DISCUSSION

PC is a protrusion abnormality of the anterior chest wall due to overgrowth of the costal cartilages.

The pathophysiology of PC is controversial; however, the most accepted theory is the defective growth of costal cartilage. This deformity is usually asymptomatic but can present with exertional dyspnoea, decreased endurance and increased respiratory tract infections.

It may present as an isolated anomaly or associated with another skeletal anomaly, other syndromes or congenital heart diseases [1].

The first-line therapy of PC is non-surgical correction with bracing. Other surgical options are performed for cosmetic reasons or for treating cardiopulmonary complications and preventing progressive postural deformities.

Surgical intervention was done traditionally by the Ravitch technique. Currently, minimal access repair of pectus carinatum is the favoured surgical option; specifically, the Abramson technique [2].

Scheuermann (juvenile) kyphosis is a progressive structural deformity of the thoracic spine that occurs in early adolescence and continues to progress with growth. It is defined by anterior wedging of at least 5° of 3 or more adjacent thoracic vertebral bodies. It is more common in males, leading to back pain [3].

Correcting severe cases usually requires bracing and possibly surgery. Typically, orthopaedic surgeons delay surgery until the child reaches his full height and the surgery is often recommended when the curve is >70° [3].

The adopted surgical approaches include anterior, posterior or combined approaches. Compared to the combined approach, the posterior-only approach reduces blood loss and avoids the risk of thoracotomy. However, it has higher rates of pseudoarthrosis [4].

The surgical correction of PC via Abramson procedure and for kyphosis via posterior approach, separately, has excellent long-term outcomes and one-stage procedure means less surgery and less pain.

The combined correction of PC and kyphosis in this report had shown satisfying results with almost complete correction of deformities without significant complications.

## CONCLUSION

PC and kyphosis are uncommon anomalies, presenting in combination is an extremely rare occasion; for which combined correction was not reported earlier in the literature. In this report, a child with both deformities had undergone a successful combined surgical correction of PC using the Abramson procedure and kyphosis using Ponte osteotomy. Results were pleasant with an uneventful postoperative course.

**Conflict of interest:** none declared.

### Reviewer information

Interactive CardioVascular and Thoracic Surgery thanks Toru Bando, Madhuri Rao and the other anonymous reviewers for their contribution to the peer review process of this article.
